# Supplementary material for: Interneuronal mechanisms for learning-induced switch in a sensory response that anticipates changes in behavioral outcomes
Source: Curr Biol. 2021 Apr 26;31(8):1754–1761.e3. doi: 10.1016/j.cub.2021.01.072 (PMC8082272; doi:10.1016/j.cub.2021.01.072)
Supplement: Document S1. Figures S1 and S2 [file mmc1.pdf]

Current Biology, Volume *31*

Supplemental Information

Interneuronal mechanisms for learning-induced switch in a sensory response that anticipates changes in behavioral outcomes

Zsolt Pirger, Zita László, Souvik Naskar, Michael Crossley, Michael O'Shea, Paul R. Benjamin, György Kemenes, and Ildikó Kemenes

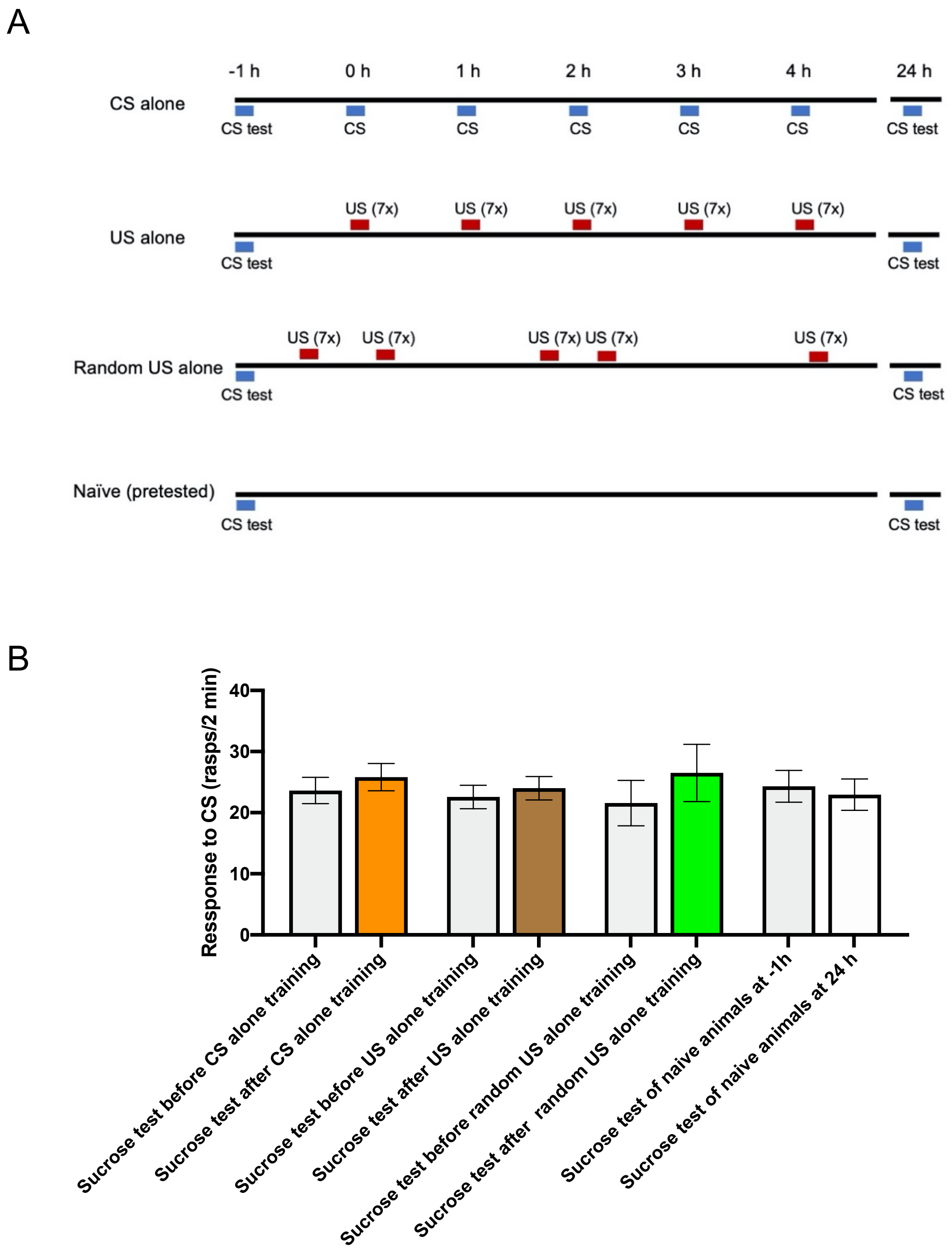

Figure S1. Non-associative training protocols do not reduce the innate behavioural response to a food stimulus. Related to Figure 1. A. The experimental groups and protocols used in the experiments. In each of the 5 CS (conditioned stimulus) alone trials only sucrose was given to the animals, in each of the 5 US (unconditioned stimulus) alone and random US alone trials, respectively, only a series of 7 strong tactile stimuli were delivered at 15 s intervals to the head of the animals. B. After the non-associative protocols, neither group shows a significantly reduced feeding response to sucrose compared to their responses before training (unpaired t-tests, each p value > 0.05). The naïve group that was pre-tested with sucrose at the same time as the other three groups (-1h) does not show a change in its response at 24h either. Graphs show means ± standard error of means (SEM). ANOVA for the CS alone, US alone, random US alone and Naïve 24 h test data: $F[3, 56] = 0.31$, $p = 0.82$ (n.s.).

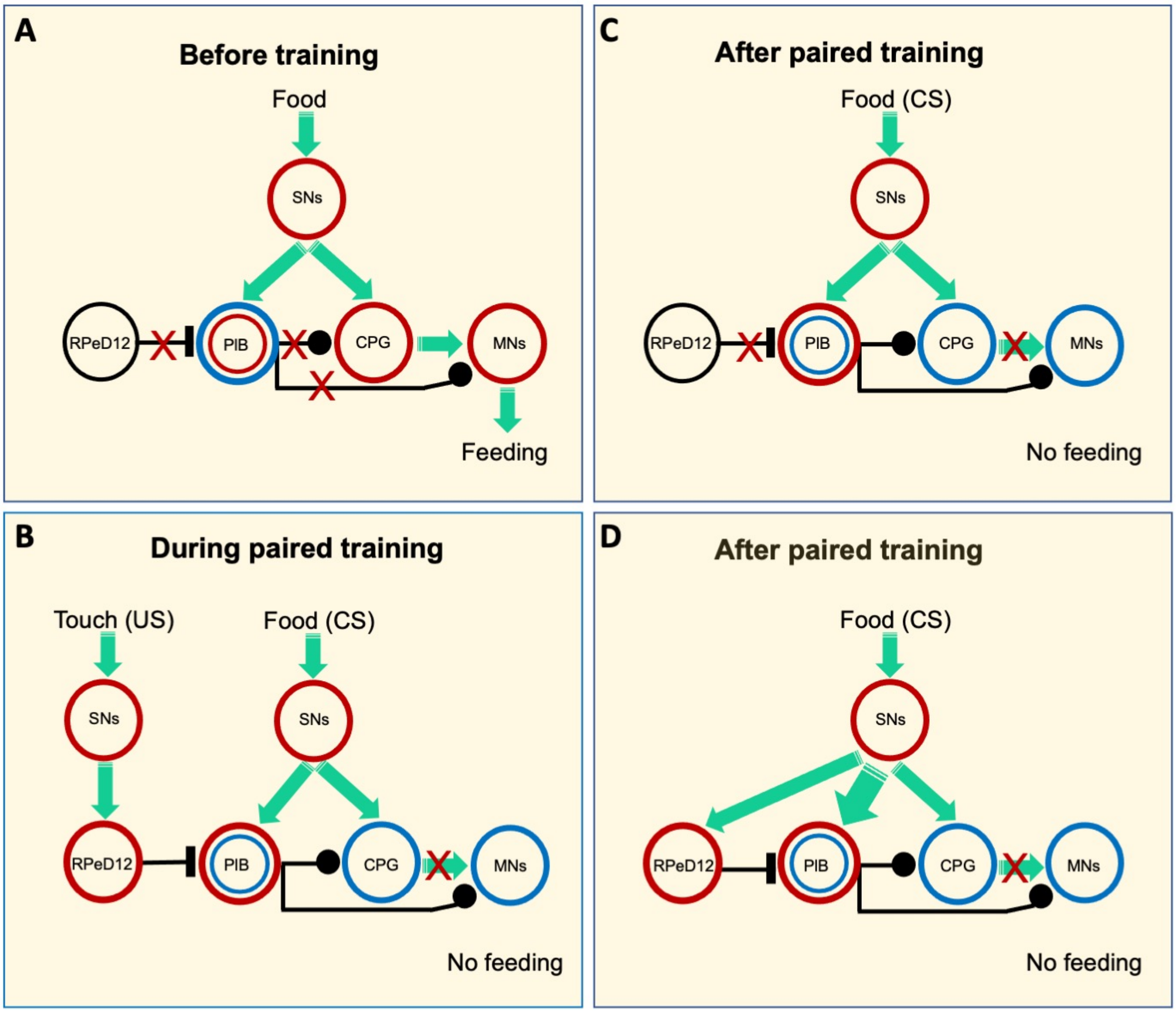

Figure S2. Simplified circuit diagrams of possible interneuronal mechanisms of conditioned inhibition of feeding by sucrose. Related to Figure 3.

A. As indicated by the red outlines, in naïve animals (before training), sucrose, a powerful food stimulus, excites the feeding central pattern generator (CPG) network[S1], which in turn activates motoneurons (MNs) of the feeding system that generate the three-phase feeding motor pattern[S2]. In the absence of the sucrose stimulus, the pleural-buccal interneuron PIB has a tonic inhibitory control over the feeding network, including the CPG and motoneurons[S3]. This tonic inhibition is temporarily suppressed (red crosses) due to the hyperpolarizing effect of the food stimulus on this interneuron (see Figures 2A and 2B), indicated by the blue outline of the circle representing PIB. The smaller red circle inside the blue one indicates the baseline level of PIB membrane potential/firing rate in naïve animals before the application of sucrose. The identified right pedal 12 withdrawal interneuron (RPeD12), which forms a strong chemical excitatory monosynaptic connection with PIB[S4], does not play a role in the circuit events of food-induced activation of the feeding motor program (indicated by the black outline of the circle representing RPeD12 and the crossed out black line with the solid black rectangle).

B. During paired training with sucrose as the CS and multiple series of strong tactile stimuli as US (see Figure 1A), RPeD12 is strongly excited by the touch US, as indicated by the red outline of the circle representing this interneuron. In turn, RPeD12 excites PIB, which will then inhibit the feeding network. The smaller blue circle indicates the hyperpolarized level of PIB membrane potential in naïve animals during the application of sucrose (the food CS) in each

trial (see Figure 1A), whereas the red outline indicates the excitation of PIB by the US-activated RPeD12. The strong excitatory input from RPeD12 to PIB overcomes the background inhibition by the sucrose-driven sensory input and results in the net depolarization of PIB. Note that potential sites of CS - US co-incidence detection in this circuit are the PIB and the CPG. However, it cannot be ruled out that terminals of the CS sensory pathway presynaptic to PIB can also be modulated during paired training.

C. One hypothetical scenario is that after paired training, there is a learning-induced plastic change in the electrical properties of PIB, for example, a persistent hyperpolarization of its baseline membrane potential level before the application of sucrose, indicated by the smaller blue circle within the red circle representing the PIB depolarized by the CS. The shift of the PIB mebrane potential to a more negative level compared to the naïve level may lead to the pre-training hyperpolarizing response to sucrose (indicated by the blue outline in A) being reversed into a depolarizing one (indicated by the red outline in C), removing the suppression of PIB's inhibitory effect on the feeding network.

D. One alternative hypothesis is that after paired training, the now aversive conditioned food stimulus sucrose also excites RPeD12, which in turn further excites PIB, which prevents the activation of the feeding network. This latter hypothesis is based on the finding that in naïve animals RPeD12 is strongly excited by aversive tactile stimulation of the head, so it seemed possible that when, after paired training, the initially strongly appetitive sucrose stimulus evokes a strong aversive response in the feeding system, this is mediated by RPeD12. We cannot rule out either that plastic changes leading to a persistent strengthening of the CS to PIB pathway (thicker green arrow connecting the sensory neurons (SNs) to PIB) also contribute to the sucrose-induced excitation of PIB after training.

The simplified diagrams in this figure only show the identified monosynaptic excitatory (black lines with solid squares) and inhibitory (black lines with solid circles) connections between RPeD12 and PIB[S4], and PIB and the CPG as well as the feeding motoneurons[S3], respectively. All other known and putative direct or indirect inputs are indicated by the green arrows.

Supplemental References

S1. Kemenes, G., Staras, K., and Benjamin, P.R. (2001) Multiple types of control by identified interneurons in a sensory-activated rhythmic motor pattern. J Neurosci *21*, 2903-2911.

S2. Rose, R.M., and Benjamin, P.R. (1979). The relationship of the central motor pattern to the feeding cycle of *Lymnaea stagnalis*. J Exp Biol *80*, 137-163.

S3. Alania, M., Sakharov, D.A., and Elliott, C.J. (2004). Multilevel inhibition of feeding by a peptidergic pleural interneuron in the mollusc *Lymnaea stagnalis*. J Comp Physiol A Neuroethol Sens Neural Behav Physiol *190*, 379-390.

S4. Pirger, Z., Crossley, M., Laszlo, Z., Naskar, S., Kemenes, G., O'Shea, M., Benjamin, P.R., and Kemenes, I. (2014). Interneuronal mechanism for Tinbergen's hierarchical model of behavioral choice. Curr Biol *24*, 2018-2024.
