# Supplementary material for: Interneuronal mechanisms for learning-induced switch in a sensory response that anticipates changes in behavioral outcomes
Source: Curr Biol. 2021 Apr 26;31(8):1754–1761.e3. doi: 10.1016/j.cub.2021.01.072 (PMC8082272; doi:10.1016/j.cub.2021.01.072)
Supplement: Document S2. Article plus Supplemental information [file mmc2.pdf]

Report

Current Biology

Interneuronal mechanisms for learning-induced switch in a sensory response that anticipates changes in behavioral outcomes

Graphical Abstract

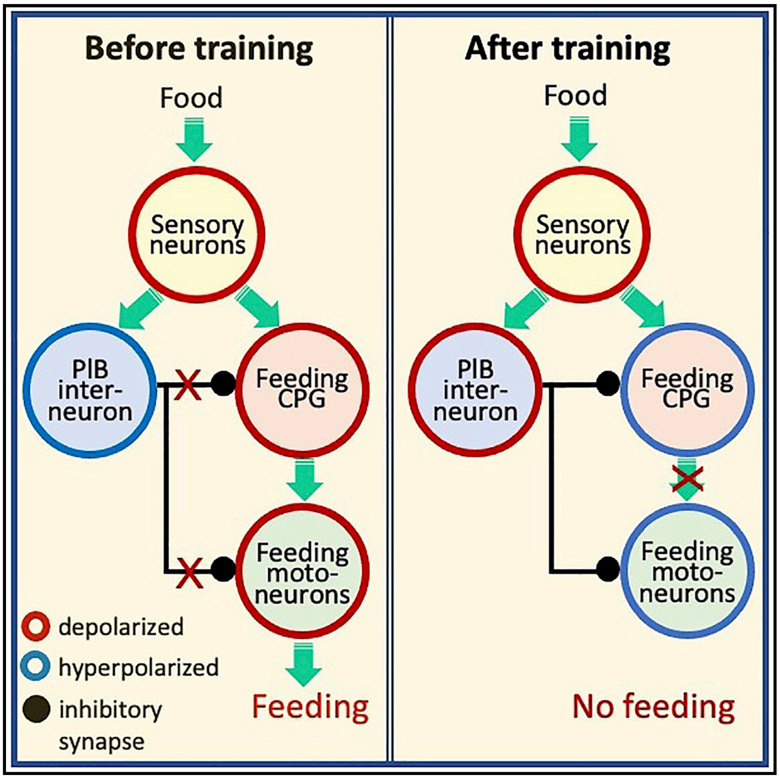

Authors

Zsolt Pirger, Zita László, Souvik Naskar, ..., Paul R. Benjamin, György Kemenes, Ildikó Kemenes

Correspondence

i.kemenes@sussex.ac.uk

In Brief

Pirger et al. demonstrate that persistent modulation of an inhibitory interneuron extrinsic to the feeding system can switch the behavioral response so that a previously salient food stimulus elicits an aversive response. Anticipation of punishment associated with food induces excitation of an interneuron that inhibits the feeding network.

Highlights

- Anticipation of an aversive outcome reverses the behavioral response to food.
- The switching mechanism relies on an interneuron extrinsic to the feeding network.
- Aversive learning causes persistent physiological change in this interneuron.

Pirger et al., 2021, Current Biology *31*, 1754–1761
April 26, 2021 © 2021 The Authors. Published by Elsevier Inc.
https://doi.org/10.1016/j.cub.2021.01.072

CellPress
OPEN ACCESS

Current Biology

Report

Interneuronal mechanisms for learning-induced switch in a sensory response that anticipates changes in behavioral outcomes

Zsolt Pirger,[1,2] Zita László,[1,2] Souvik Naskar,[1,3] Michael Crossley,[1,5] Michael O'Shea,[1,5] Paul R. Benjamin,[1,5] György Kemenes,[1,4,5] and Ildikó Kemenes[1,5,6,*]
[1]Sussex Neuroscience, School of Life Sciences, University of Sussex, Brighton BN1 9QG, UK
[2]Present address: Adaptive Neuroethological Research Group, Department of Experimental Zoology, Balaton Limnological Institute, Centre for Ecological Research, Tihany 8237, Hungary
[3]Present address: Department of Life Sciences, Faculty of Natural Sciences, Imperial College London, London SW7 2AZ, UK
[4]Twitter: @GeorgeKemenes
[5]Twitter: @SussexNeuro
[6]Lead contact
*Correspondence: i.kemenes@sussex.ac.uk
https://doi.org/10.1016/j.cub.2021.01.072

SUMMARY

Sensory cues in the natural environment predict reward or punishment, important for survival. For example, the ability to detect attractive tastes indicating palatable food is essential for foraging while the recognition of inedible substrates prevents harm. While some of these sensory responses are innate, they can undergo fundamental changes due to prior experience associated with the stimulus. However, the mechanisms underlying such behavioral switching of an innate sensory response at the neuron and network levels require further investigation. We used the model learning system of *Lymnaea stagnalis*[1-3] to address the question of how an anticipated aversive outcome reverses the behavioral response to a previously effective feeding stimulus, sucrose. Key to the switching mechanism is an extrinsic inhibitory interneuron of the feeding network, PIB (pleural buccal[4,5]), which is inhibited by sucrose to allow a feeding response. After multi-trial aversive associative conditioning, pairing sucrose with strong tactile stimuli to the head, PIB's firing rate increases in response to sucrose application to the lips and the feeding response is suppressed; this learned response is reversed by the photoinactivation of a single PIB. A learning-induced persistent change in the cellular properties of PIB that results in an increase rather than a decrease in its firing rate in response to sucrose provides a neurophysiological mechanism for this behavioral switch. A key interneuron, PeD12 (Pedal-Dorsal 12), of the defensive withdrawal network[5,6] does not mediate the conditioned suppression of feeding, but its facilitated output contributes to the sensitization of the withdrawal response.

RESULTS

Switching of the behavioral feeding response to sucrose after aversive conditioning

As predicted by the model that suggests behavioral responses to sensory stimuli depend on prior learning experiences, aversive conditioning in *Lymnaea* results in a selective impairment of the feeding response to sucrose, an innate feeding stimulus. We obtained the behavioral evidence by developing a 5-trial aversive conditioning paradigm (Figure 1A) in which in each trial, sucrose was the conditioned stimulus (CS) and a series of aversive tactile stimuli to the head served as the unconditioned stimulus (US) that evoked a whole-body withdrawal response. Unpaired and naive groups were used as the primary controls (Figure 1A), but a number of other control groups (CS alone, US alone, random US alone and a naive group pre-tested with the CS prior to the commencement of training in the paired group and tested again at the same time as the other groups at 24 h post-training) also were used to fully rule out the possibility of non-associative learning (Figure S1A).

At 24 h post-training, the CS+US paired group of animals showed a significantly reduced feeding response compared to the unpaired and naive group, and also compared to its own pre-training feeding response to sucrose (Figure 1B, statistics in legend). There was no significant difference between the pre- and post-training feeding responses to sucrose in the unpaired and naive group (Figure 1B) or in any of the other control groups used in the experiments (Figure S1B, statistics in legend), confirming that the change in the CS+US paired group was the result of associative learning. It was also important to confirm that the reduction of the behavioral response after aversive conditioning is specific to the sucrose stimulus. Another salient feeding stimulus, fresh cucumber juice, was applied 24 h after aversive training with sucrose as the CS but there were no reductions in the feeding response (Figure 1B, statistics in legend).

This is an open access article under the CC BY license (http://creativecommons.org/licenses/by/4.0/).

CellPress
OPEN ACCESS

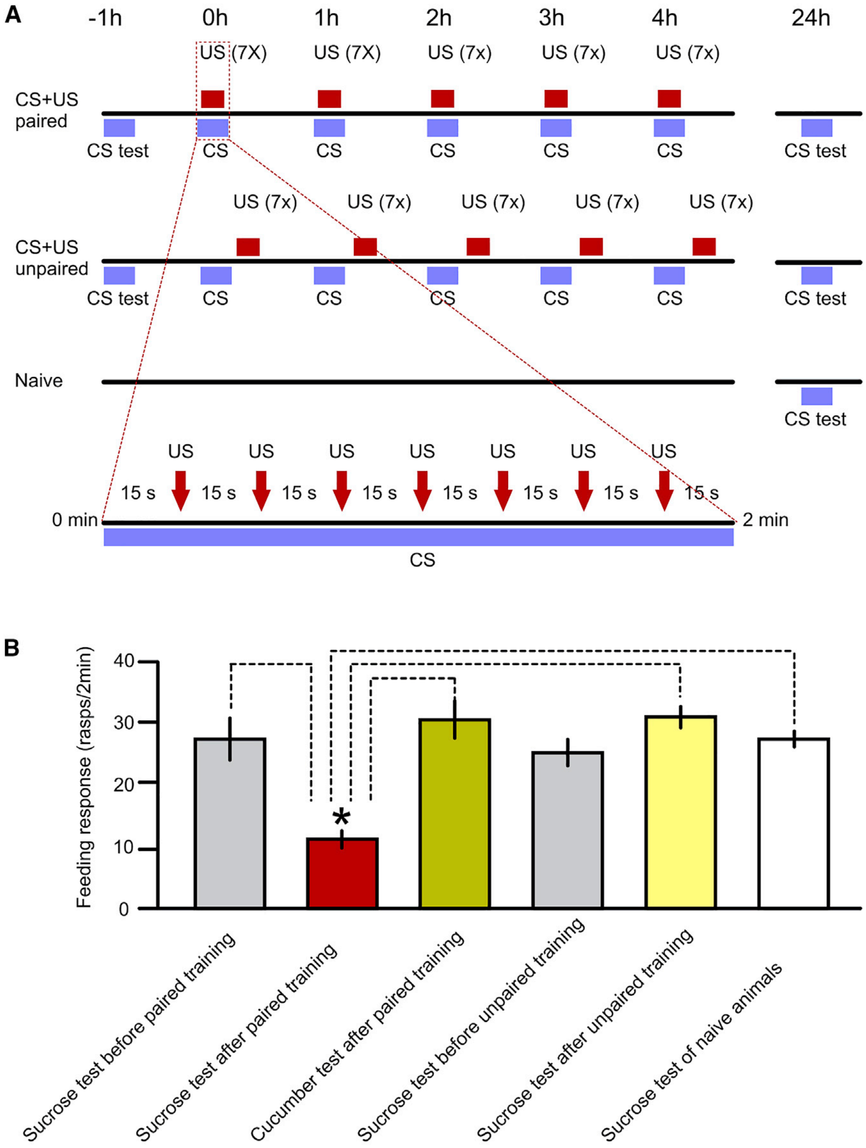

Figure 1. Aversive classical conditioning reduces the innate behavioral response to a food stimulus

(A) The experimental groups and protocols used in the behavioral experiments. In each of the 5 paired and unpaired episodes of trials the CS (conditioned stimulus) was sucrose, the series of US (unconditioned stimuli) was 7 strong tactile stimuli delivered using a hand-held probe with a tip made of a tooth-pick at 15 s intervals to the head of the animals, which evoked whole-body withdrawal. In the paired trials (see insert), the CS to first US interval was 15 s; in the unpaired trials it was 10 min.

(B) After aversive classical conditioning, only the CS+US paired group shows a significantly reduced feeding response to sucrose, a highly salient food stimulus in control animals (also compare to additional controls in Figure S1). Graphs show means ± standard error of means (SEM). Asterisk indicates significance detected by the pairwise and multiple comparisons made after the ANOVA. ANOVA for the CS+US paired, CS+US unpaired and Naive 24 h test data: $F[3, 50] = 19.07$, $p < 0.0001$; Tukey's tests: CS+US paired group ($n = 12$) versus CS+US unpaired ($n = 12$) and Naive group ($n = 18$), $p < 0.0001$ for both; CS+US unpaired group versus Naive group, $p = 0.84$). Aversive conditioning with sucrose as the CS did not affect the feeding response to a different food stimulus, cucumber juice. Although the group of aversively conditioned animals shows a significant reduction compared to their pre-training feeding response to sucrose (unpaired t test, $t = 4.36$, $df = 22$, $p = 0.0003$), it shows the same high level of feeding response to the cucumber juice as it did to sucrose before training (unpaired t test, $t = 0.65$, $df = 22$, $p = 0.5252$) and as the unpaired and naive control animals after training (ANOVA, $F[2, 39]$ $p = 0.63$). The cucumber test data are significantly higher than the sucrose test after paired training data (unpaired t test, $t = 5.4$, $df = 2$, $p < 0.0001$).

Interneuronal mechanism of feeding inhibition after aversive conditioning

We reasoned that a learning-induced long-term reversal of the hyperpolarizing response of the PIB interneurons to sucrose-activated sensory inputs (Figures S2A and S2B) underlies the observed behavioral changes induced by aversive conditioning. PIB has an inhibitory effect on the feeding CPG and motoneurons[4,5]. In naive animals, this inhibition is weakened in the presence of sucrose, allowing the activation of the feeding network[5]. The hyperpolarizing effect of sucrose that underlies the removal of tonic inhibition of the feeding network by PIB needs to be reversed for sucrose to be able to inhibit rather than activate feeding in aversively conditioned animals. Alternative hypotheses for the interneuronal mechanisms involved in a post-training reversal of sucrose-induced hyperpolarization of PIB are outlined in Figures S2C and S2D). Using a semi-intact preparation, we carried out intracellular electrophysiological recordings of PIB and simultaneously recorded feeding ingestion movements of the feeding apparatus[7] (the buccal mass) to monitor changes in the neural and muscular activity following sucrose application to the lips. Comparisons were made of the changes in membrane potentials and firing frequencies of the PIB neurons in response to sucrose in semi-intact lip-CNS preparations made from snails that had been subjected either to paired or unpaired behavioral protocols or were from naive animals (n = 10 in each group). In preparations from naive and unpaired animals, sucrose application to the lips hyperpolarized PIB and consequently reduced its spontaneous tonic firing (to reduce its inhibitory effects on the neurons of the feeding network), and this was accompanied by an increase in the frequency of muscular contractions of the buccal mass (Figures 2A and 2B) that underlie ingestive feeding behavior in the intact animal[7]. In contrast, in preparations from aversively trained animals the PIB cell showed a marked depolarization and a consequent increase in its tonic firing rate (to increase its inhibitory effects on the feeding network) during the application of sucrose to the lips, accompanied by a cessation

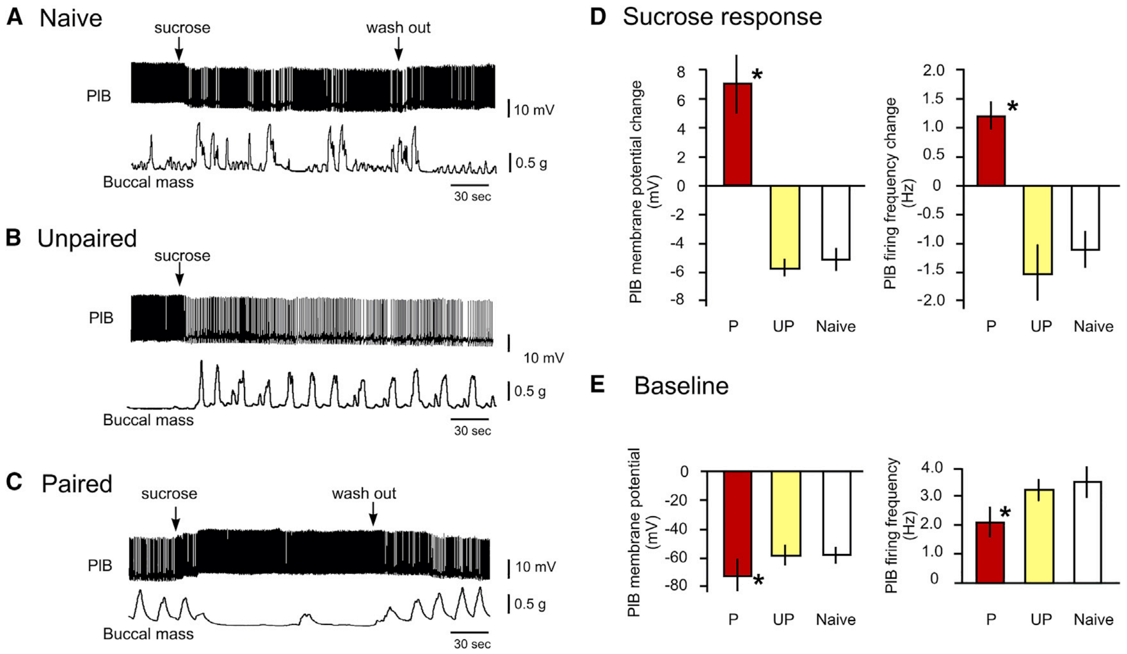

Figure 2. Aversive classical conditioning reverses the electrophysiological response of an identified modulatory interneuron, PIB, to a food stimulus

(A and B) In typical semi-intact preparations from naive and unpaired control animals, the application of sucrose to the lips results in the hyperpolarization and resulting reduction of the firing rate of the modulatory interneuron PIB and the triggering of rhythmic contractions of the buccal mass. The Unpaired trace in (B) illustrates that if the sucrose stimulus is not removed from the sensory areas, PIB remains hyperpolarized with a correspondingly lower spike frequency, and rhythmic feeding activity continues.

(C) Representative example of a semi-intact preparation from a CS+US paired animal where the application of sucrose to the lips results in the depolarization and resulting increase of the firing rate of PIB and the cessation of the spontaneous rhythmic contractions of the buccal mass.

(D) Statistical comparisons of the sucrose-evoked electrophysiological responses of PIBs in preparations from CS+US paired and control (unpaired [UP] and naive) animals. The measurements were taken using Spike 2 in the 30 s period before and after the application of sucrose and the change was computed as the numerical difference between the pre- and post-sucrose data. When compared among the naive ($n = 10$), unpaired ($n = 10$), and paired groups ($n = 10$), both the changes in PIB membrane potential and firing rate in response to sucrose are consistently in the opposite direction and highly significantly different in the paired versus the other two groups (ANOVA for membrane potential change data: $F[2, 27] = 24.69$, $p < 0.0001$, Tukey's: $p < 0.001$ for both comparisons. ANOVA for spike frequency change data: $F[2, 27] = 16.23$, $p < 0.0001$), Tukey's: $p < 0.05$ for both comparisons). Neither the membrane potential nor the spike frequency changes are significantly different between the naive and unpaired group (Tukey's: $p = 0.84$ and 0.42, respectively).

(E) Statistical comparisons of baseline (intrinsic) electrical properties (membrane potential and firing frequency in the absence of a sucrose stimulus) of PIBs in preparations from CS+US paired (P) and control (unpaired (UP) and naive) animals. PIBs from CS+US trained animals have a significantly more hyperpolarized baseline membrane potential and lower baseline spike frequency compared to PIBs from both naive and unpaired animals. ANOVA for baseline membrane potential data: $F[2, 27] = 4.40$, $p < 0.03$, Tukey's for both comparisons: $p < 0.05$, Tukey's for unpaired and naive comparison: $p = 0.99$. ANOVA for baseline spike frequency data: $F[2, 27] = 4.72$, $p < 0.02$, Tukey's for both comparisons: $p < 0.05$, Tukey's for unpaired and naive comparison: $p = 0.98$.

Graphs in (D) and (E) show means ± standard error of means (SEM). Asterisks indicate statistical significance of at least $p < 0.05$.

of spontaneous rhythmic buccal mass movements (Figure 2C, statistical comparisons between the three groups are shown in Figure 2D). Spontaneous buccal mass contractions were observed in the lip-CNS-buccal mass preparations from aversively trained animals (example in Figure 2C), whereas the same type of preparations made from snails that had been subjected either to the unpaired protocol or were from naive animals showed no or occasional contractions of the buccal mass (examples in Figures 2A and 2B). These results support the conclusion that by increasing PIB's firing rate, sucrose is now strongly activating the inhibitory PIB to feeding circuit pathway, known to be involved in the suppression of feeding[4].

Further analysis of the data revealed a significant difference between the baseline membrane potential (in the absence of sucrose presentation) and consequent baseline spike frequency levels of the PIBs in preparations from paired versus naive and unpaired control animals, respectively, 24 h after training. PIBs from the CS+US trained animals had significantly more hyperpolarized baseline membrane potential and lower baseline spike frequency compared with those from naive and unpaired groups of animals (Figure 2E, statistics in legend). This suggests that aversive conditioning changes the intrinsic cellular properties of the PIBs to control the firing rate of this inhibitory interneuron; with increased background hyperpolarization, the excitatory response in the presence of sucrose could be more robust (as inactivation of sodium and calcium channels would be reduced). This possibility however needs to be tested in future experiments.

Photo-inactivation of a single PIB removes the effects of aversive conditioning yielding strong evidence that the PIB interneurons are the main locus for the learning-induced changes in the sucrose response. Photo-inactivation of carboxyfluorescein-filled

CellPress
OPEN ACCESS

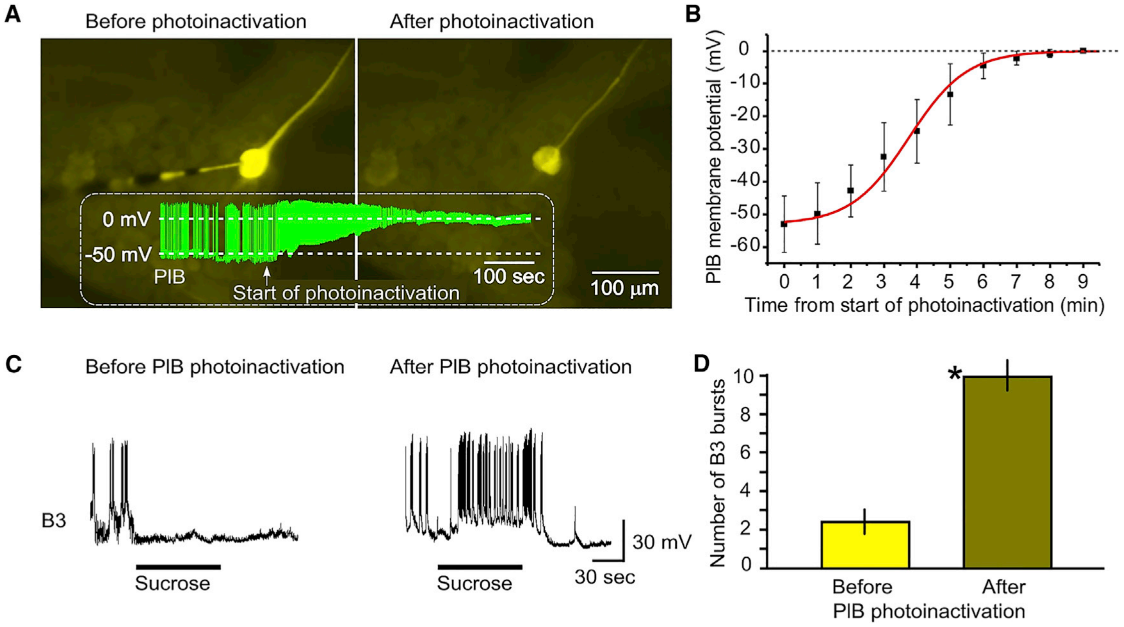

Figure 3. Photoinactivation of PIB converts the paired-preparation phenotype to a naive one

(A and B) The temporal progression of the loss of the electrical properties (firing and membrane potential) of carboxyfluorescein-filled PIB neurons during photoinactivation (example trace of a PIB being photoinactivated shown in green in (A), white arrow indicates onset of photoinactivation).

(C) Left, cessation of spontaneous and absence of sucrose-induced fictive feeding cycles recorded on a B3 feeding motoneuron in a semi-intact preparation from a CS+US paired animal before PIB photoinactivation (see hypothetical scenario in Figure S2C). Right, after PIB photoinactivation in the same preparation, the feeding motoneuron shows fictive feeding cycles in response to sucrose applied to the lips.

D. Statistical comparison of the number of fictive feeding bursts recorded in B3 during the application of sucrose. Graphs show means ± standard error of means (SEM). n = 8 preparations, paired t test: $p < 0.0003$ (asterisk indicates significance).

PIB interneurons showed a progressive reduction in the resting membrane potential in aversively trained animals from −53 ± 8 mV to 0 mV within 7 min, leading to a gradual loss of PIB spike activity (Figures 3A and 3B). A previous study[8] showed that there is electrical coupling between the paired PIBs sufficient in strength for them to act as a single unit. Thus, the photoinactivation of one PIB will automatically block the spiking in the contra-lateral PIB homolog and prevent inhibition of feeding by sucrose. This is similar to what was demonstrated previously in photoinactivation experiments targeting the also electrotonically coupled pair of N1M feeding CPG interneurons, which showed that the photoablation of a single N1M can block the activation of the feeding rhythm by a higher-order modulatory neuron[9].

Rhythmic motoneuronal feeding activity following application of sucrose was recorded before and after photo-inactivation (Figures 3C and 3D). Prior to PIB photoinactivation, the application of sucrose to the lips inhibited the spontaneous fictive feeding activity in the B3 feeding motoneuron and failed to trigger rhythmic feeding activity (Figure 3C, left). After photoinactivation, the same preparations regained their ability to respond to the sucrose stimulus with an increased number of feeding motoneuronal bursts (Figure 3C, right), a pattern of firing that underlies cyclical feeding in the intact animal. A comparison of the fictive feeding cycles recorded in B3 after the application of sucrose showed a significant difference between the feeding rates before and after PIB had been photo-inactivated (Figure 3D, statistics in legend).

The withdrawal response interneuron PeD12 is not a pathway for the inhibitory sucrose responses following aversive conditioning but its facilitated output contributes to the sensitized withdrawal response to mild touch

Although the photoinactivation experiments confirmed that PIB was necessary for the conditioned suppression of feeding response in response to the CS, we could not rule out that one or more other interneurons were driving its strong depolarization when sucrose was applied to the lips. Our previous study showed that the withdrawal response interneuron PeD12 is a key element in touch-induced inhibition of feeding[5]. Aversive touch excites PeD12 and via a monosynaptic excitatory pathway increases the tonic firing of PIB leading to the suppression of feeding. We reasoned that following CS+US paired training, the now aversive sucrose stimulus could activate PeD12 in the same way aversive touch activates it in preparations from naive animals[5]. We set up semi-intact lip-CNS preparations from CS+US trained, unpaired and naive control animals and co-recorded PeD12 with a B3 or B4 feeding motoneuron to establish if PeD12 responded to the sucrose CS after aversive classical conditioning and could therefore be a source of the sucrose-triggered excitatory PIB response after paired training (hypothesis outlined in Figure S2C). We found that although the fictive feeding response was suppressed in preparations from CS+US paired animals (example in top traces of Figure 4A), PeD12 did not respond to the sucrose CS with increased firing and so plays no role in the blocking of feeding and neither

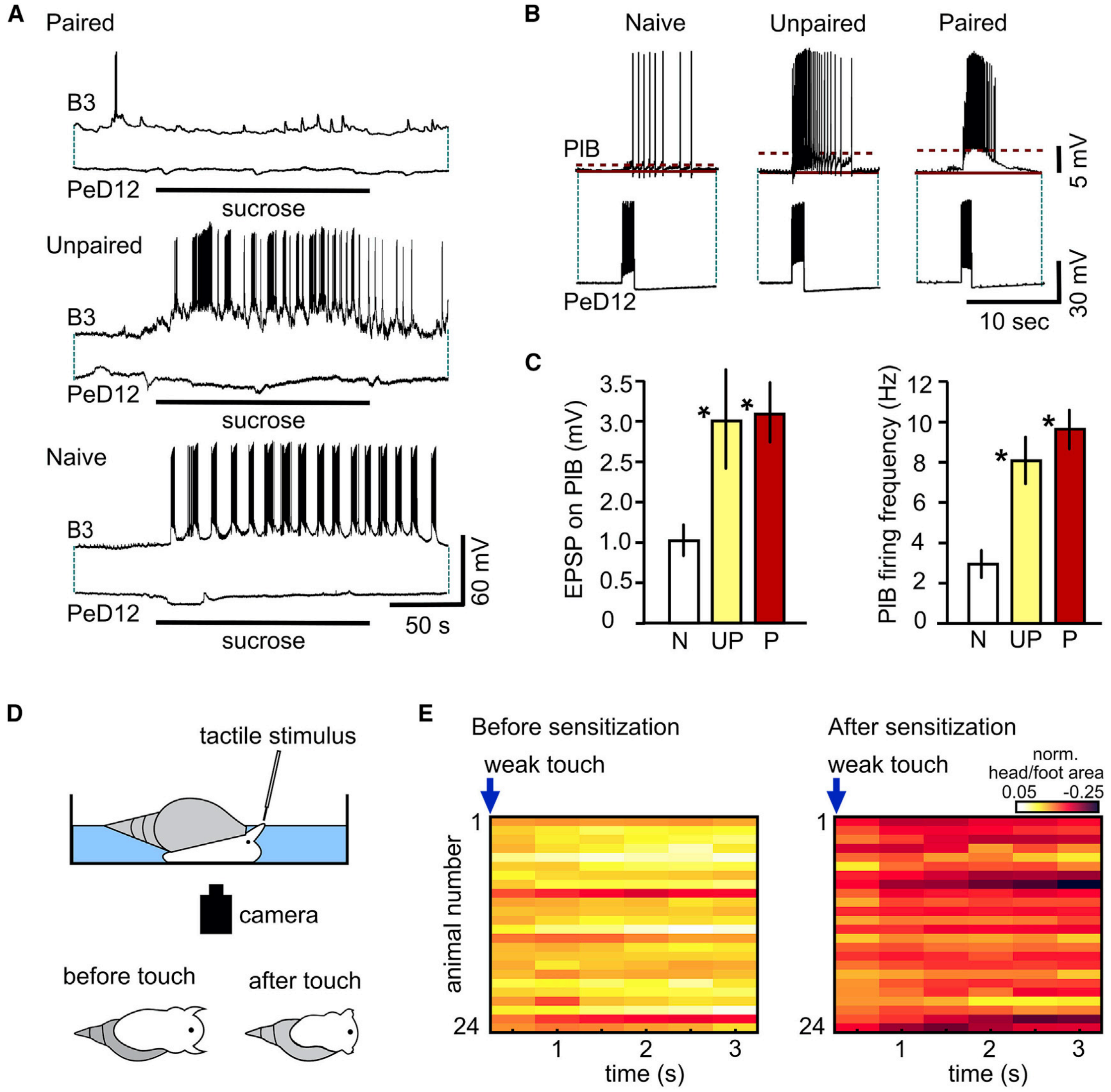

Figure 4. The withdrawal interneuron PeD12 is not involved in the conditioned aversive response but plays a role in sensitization

(A) Top two traces: simultaneous recordings of the feeding motoneuron B3 and the withdrawal interneuron PeD12 in a preparation made from a CS+US paired animal showing the lack of a fictive feeding response in B3, in the absence of sucrose-triggered activation of PeD12. Middle and bottom traces: sucrose activates fictive feeding in preparations from an unpaired and a naive animal, respectively. As expected, the withdrawal interneuron, PeD12, shows no activation from the application of sucrose, a rewarding food stimulus.

(B) Square-pulse triggered bursts of action potentials in PeD12 evoke facilitated excitatory postsynaptic responses in PIBs in preparations from both a CS+US paired and an unpaired animal compared to a preparation from a naive animal. In these experiments the PIBs were hyperpolarized briefly to the same membrane potential level (−80 mV) via the recording electrode to temporarily stop their tonic firing activity while testing their responses to the PeD12 bursts.

(C) Statistical comparisons of the PeD12-triggered PIB EPSP peak amplitudes and spike frequencies (the latter calculated from the number of spikes in the first 3 s of the PIB bursts) in preparations from naive (N) versus unpaired (UP) and CS+US paired (P) animals. Graphs show means ± standard error of means (SEM). Asterisks indicate significance of at least $p < 0.05$ (compared against the naive data). Twenty-four h after training, an artificially triggered burst of PeD12 action potentials resulted in a significant increase in both the postsynaptic depolarization and spike frequency recorded in the PIB in preparations from CS+US paired ($n = 17$) as well as unpaired animals ($n = 13$) compared to naive controls ($n = 17$) (ANOVAs: $F[2, 44] = 9.17$, $p < 0.0005$ (EPSP size), $F,[2, 44]$ $p < 0.0001$ (spike frequency); Tukey's multiple comparisons tests: $p < 0.001$ for both Naive versus Paired and Naive versus Unpaired, for both EPSP size and spike frequency, $p = 0.98$ for Unpaired versus Paired (EPSP size) and $p = 0.600$ for Unpaired versus Paired (spike frequency).

(D) Cartoon of the behavioral sensitization analysis. Animals were placed in a Petri dish and a weak tactile stimulus applied to the tentacle, which evoked a withdrawal of the tentacles as well as the head/foot complex. Animals were videoed from below and analyzed offline. One second before the stimulus, the total head/foot area of the animal was measured, and again at 0.5, 1, 1.5, 2, 2.5, and 3 s after the stimulus. The withdrawal response was measured as a decrease in total area, as illustrated in the cartoon of a snail before and after touch.

(E) Heat plots of the head/foot area of the same animals ($n = 24$) before and after sensitization. Data was normalized to the pre-stimulus condition. At each post-stimulus time point, the mean withdrawal response to the weak tactile stimulus was significantly stronger when the same snails were tested

(legend continued on next page)

does it play a role in the activation of feeding by sucrose in preparations from CS+US unpaired or naive animals, as shown in the examples in the middle and bottom traces of Figure 4A.

Simultaneous recordings from PeD12 and PIB (examples in Figure 4B) revealed that the strength of the post-synaptic excitatory response in PIB to artificially triggered PeD12 bursts was significantly increased in preparations from both paired and unpaired animals compared with naive controls (Figure 4C, statistics in legend). This suggested that even though PeD12 was not recruited by the aversive training to mediate the conditioned excitatory effect of sucrose on PIB, its excitatory synaptic output was facilitated after both paired and unpaired aversive training. Based on this finding, we predicted that the animals would be sensitized by the strong tactile stimulation alone that triggers bursts of action potentials in RPeD12 to drive a whole-body withdrawal response[5].

Evidence for sensitization was obtained in behavioral experiments. Series of strong tactile stimuli were applied to the lips in the same temporal pattern as during paired or unpaired aversive conditioning (see Figure 1A) but without the sucrose CS. The withdrawal response to a weak tactile stimulus was tested 24 h after the training and compared with pre-training behavioral responses to the same weak tactile stimulus using quantitative analysis of videos that, unknown to the observer, were either from pre- or post-training animals. These experiments revealed that animals showed a significantly greater withdrawal 24 h after sensitization training compared to 1 h before training in response to the same weak tactile stimulus (Figures 4D and 4E, statistics in legend).

DISCUSSION

Our results conform to the general model that behavioral responses to a sensory stimulus depend on the prior experience associated with this stimulus and we provide an interneuronal mechanism for it. Aversive conditioning in *Lymnaea* results in a selective impairment of the feeding response to sucrose, an innate feeding stimulus. Strong touches to the head provided the aversive unconditioned stimuli in the conditioning experiments because they trigger a defensive withdrawal response and inhibit feeding[5]. However, unless the sucrose CS is explicitly paired with multiple series of US to the head, there is no reversal of the feeding response to sucrose. Thus, unpaired application of the CS and US, the application of the CS or US alone and random application of head touch do not lead to long-lasting alteration of the innate feeding response (see Figure 1 and Figure S1). This provides evidence that the change in the response to sucrose depends on the explicit association with aversive stimuli so that the sucrose sensory stimulus now anticipates a long-lasting future danger to the animal, rather than food. Interestingly, when sensitization training with strong electric shocks to the body wall was used in *Aplysia*, it resulted in a persistent suppression of feeding behavior as well as a sensitized defensive response to a brief, mild current pulse to the tail[10]. Based on these previous findings we would have expected to see a suppressed feeding response in both the Paired and Unpaired groups of animals in our experiments. However, we only found a reduced feeding response after paired training indicating that aversive associative and non-associative training may have different effects on the neural circuits controlling the withdrawal and feeding network.

Our earlier work[11] on aversive classical conditioning of feeding in *Lymnaea* identified the PIB modulatory interneuron as a significant part of the feeding learning circuit involved in this type of associative learning. Isolated CNS preparations were made from animals aversively conditioned with L-serine, as the appetitive chemical CS and quinine as the aversive chemical US. Electrical stimulation of the CS chemosensory neurons in their lip nerve pathway to the CNS caused a significant increase in PIB firing rate compared with naive controls, and a reduced expression of fictive feeding cycles in motor neurones of the feeding circuit, i.e., an *in vitro* correlate of the behavioral conditioned response. Although this experiment showed in principle that PIB was involved in aversive associative conditioning, the role of strong tactile stimulation linked to the inhibition of feeding[5] was not established. Therefore, in the present study we carried out an additional associative learning experiment where aversive tactile stimuli were used as the US. Application of sucrose (the CS) to the lips in semi-intact preparations from aversively conditioned animals increased PIB firing frequency and inhibited feeding via inhibitory synaptic connections with the interneurons and motoneurons of the feeding network[4], whereas in preparations from control animals that received unpaired training or no training, the opposite happened: PIB firing frequency decreased and feeding was activated in response to the sucrose stimulus. The baseline membrane potential of PIBs from aversively trained PIBs is significantly more hyperpolarized than in PIBs from both unpaired and naive control animals resulting in a significantly lower rate of tonic spike activity and a corresponding increase in ingestion feeding movements. The hyperpolarization of the PIB is long-lasting (recorded at 24 h after aversive training) and not dependent on any sensory stimulation, so we assume that there must be a persistent change in the biophysical properties of the interneuron. However, we cannot rule out that this hyperpolarization is at least partially due to a persistent tonic inhibitory input from another, so far unidentified, neuron. The subsequent depolarization of PIB by sucrose application could be a rebound effect from the hyperpolarization due to aversive conditioning because in the control groups the hyperpolarization is significantly less. Although there is an indication of an increase in excitability resulting from artificial hyperpolarization of PIB in one of our previous studies[5], this hypothesis needs to be tested by further electrophysiological experiments.

Although we ruled out RPeD12 as a source of enhanced excitatory input to PIB after training, there may be further associative changes elsewhere in the circuit, affecting the CS to PIB pathway presynaptically to PIB, which together with the intrinsic

24 h after the sensitization training, compared to when they were tested with the same stimulus 1 h before sensitization (Two-way ANOVA with RM: “Time after weak touch x Sensitization,” $F[6, 276] = 23.78$,” $p < 0.0001$; “Time after weak touch,” $F[2.2, 101.0] = 50.80$,” $p < 0.0001$; “Sensitization,” $F[1, 46] = 46.33$, $p < 0.0001$; paired sample t tests comparing the Before sensitization and After sensitization data for each individual animal at each time point: $p < 0.0001$).

mechanisms, drive continuous spiking activity in PIB, sufficient to inhibit feeding behavior (Figure S2D). However, these competing hypotheses need to be tested in further experiments.

C. elegans provided a tractable nervous system that made it possible to identify an olfactory neuron (AWC) that is responsible for avoidance learning induced switching of behavioral responses to a food stimulus[12-14]. Similar to *C. elegans*, *Lymnaea* also has a single cell type that is crucial for the switching between attraction (feeding) and avoidance (whole body withdrawal). However, in the case of *Lymnaea*, the decision is made by an interneuron (PIB) rather than a sensory neuron and importantly, its electrical properties can be altered by learning. Simultaneous recordings of intracellular activity from identified neurons of both the feeding and the withdrawal circuits revealed that increasing the firing frequency of the PIB neuron by positive current injection supressed the feeding network whereas reducing the firing by hyperpolarization led to the activation of feeding[5]. After aversive conditioning, the firing rate of the PIB increases in response to application of sucrose to the lips, demonstrating that the interneuron is capable of suppressing a strong innate behavioral response. Similar changes were observed in vertebrates where populations of inhibitory neurons in the NAc increased their firing rate after aversive conditioning causing the suppression of the feeding response[15].

Extensive work on insects concentrated on identifying the molecular and neuronal mechanisms of odor aversion and attraction. These studies highlighted that neurons with related sensory receptors and similar projections to the olfactory bulb can generate opposing behaviors. Ablation experiments in *Drosophila* found that two higher order olfactory nuclei, the lateral horn and the mushroom body, mediate innate and learned odor responses, respectively[16-18].

In conclusion, two distinctly different models for behavioral switching have been identified, an interneuronal model based on extrinsic control of behavioral motor networks and a sensory model where modifications of the pathways involved in sensory processing are re-programmed to change the behavioral response. The extrinsic nature of a controlling interneuron is a positive feature because the target behavioral circuitry is not disabled and so other types of cogent sensory stimuli positive or negative could still be operational via different sensory pathways. The sensory model allows for targeted changes in neuronal pathways that code for specific sensory cues at different levels in the nervous system that process sensory information. Our example is an interesting case of the first model. It provides insights into the cellular and synaptic details of the way that inhibition mediates sensory switching. Our results suggest that that there is an important role for tonic inhibition in behavioral control of sensory responses and we suggest that it represents a general mechanism that is central to adaptive behavioral switching in other systems[19,20].

STAR★METHODS

Detailed methods are provided in the online version of this paper and include the following:

- KEY RESOURCES TABLE
- RESOURCE AVAILABILITY
  - Lead contact
  - Materials Availability
  - Data and Code Availability
  - Experimental Model and Subject Details
- METHOD DETAILS
  - Multi-trial aversive classical conditioning
  - Sensitization training
  - Preparations for electrophysiology
  - Transducer recordings
  - Electrophysiology recordings
  - Identification of neurons
  - Dye-injection and photoinactivation
- QUANTIFICATION AND STATISTICAL ANALYSIS

SUPPLEMENTAL INFORMATION

Supplemental Information can be found online at https://doi.org/10.1016/j.cub.2021.01.072.

ACKNOWLEDGMENTS

This work was funded by the Biotechnology and Biological Sciences Research Council grants BBSRC/BB/H009906/1 (M.O.S., G.K., and P.R.B.) and BBSRC/BB/P00766X/1 (I.K., G.K., and P.R.B.) and the National Brain Research Program (Hungary) grant 2017-1.2.1-NKP-2017-00002 (Z.P.).

AUTHOR CONTRIBUTIONS

Conceptualization, Z.P., M.O.S., P.R.B., G.K., and I.K. Methodology, Z.P., G.K., I.K., and M.C. Investigation, Z.P., Z.L., S.N., and M.C. Formal Analysis, G.K., I.K., M.C., and Z.P. Writing – Original draft, Z.P., M.C., G.K., and I.K. Writing – Review and Editing, P.R.B., G.K., and I.K. Visualization, Z.P., M.C., G.K., and I.K. Supervision, I.K., G.K., and P.R.B. Project Administration, I.K. Funding acquisition, I.K., G.K., P.R.B., M.O.S., and Z.P.

DECLARATION OF INTERESTS

The authors declare no competing interests.

Received: July 14, 2020
Revised: October 31, 2020
Accepted: January 20, 2021
Published: February 10, 2021

REFERENCES

1. Kemenes, G. (2015). Dynamic Molecular Mechanisms of Memory Consolidation after Single-trial Food-reward Classical Conditioning in *Lymnaea*. In Memory Consolidation, I.E, M. Sakakibara, ed. (Nova), pp. 127–141.
2. Benjamin, P.R., and Kemenes, G. (2017). Behavioral and Circuit Analysis of Learning and Memory in Mollusks. In Learning and Memory: A Comprehensive Reference, *Volume 4*, John H. Byrne, ed. (Elsevier), pp. 427–440.
3. Fodor, I., Hussein, A.A., Benjamin, P.R., Koene, J.M., and Pirger, Z. (2020). The unlimited potential of the great pond snail, *Lymnaea stagnalis*. eLife *9*, e56962.
4. Alania, M., Sakharov, D.A., and Elliott, C.J. (2004). Multilevel inhibition of feeding by a peptidergic pleural interneuron in the mollusc *Lymnaea stagnalis*. J. Comp. Physiol. A Neuroethol. Sens. Neural Behav. Physiol. *190*, 379–390.
5. Pirger, Z., Crossley, M., László, Z., Naskar, S., Kemenes, G., O'Shea, M., Benjamin, P.R., and Kemenes, I. (2014). Interneuronal mechanism for Tinbergen's hierarchical model of behavioral choice. Curr. Biol. *24*, 2018–2024.

CellPress
OPEN ACCESS

6. Ferguson, G.P., and Benjamin, P.R. (1991). The whole-body withdrawal response of *Lymnaea stagnalis*. I. Identification of central motoneurones and muscles. J. Exp. Biol. *158*, 63–95.
7. Rose, R.M., and Benjamin, P.R. (1979). The relationship of the central motor pattern to the feeding cycle of *Lymnaea stagnalis*. J. Exp. Biol. *80*, 137–163.
8. Alania, M., and Sakharov, D.A. (2000). Morphology and physiology of pleural-to-buccal neurons coordinating defensive retraction with feeding arrest in the pond snail *Lymnaea stagnalis*. Acta Biol. Hung. *51*, 197–203.
9. Kemenes, G., and Elliott, C.J. (1994). Analysis of the feeding motor pattern in the pond snail, *Lymnaea stagnalis*: photoinactivation of axonally stained pattern-generating interneurons. J. Neurosci. *14*, 153–166.
10. Acheampong, A., Kelly, K., Shields-Johnson, M., Hajovsky, J., Wainwright, M., and Mozzachiodi, R. (2012). Rapid and persistent suppression of feeding behavior induced by sensitization training in *Aplysia*. Learn. Mem. *19*, 159–163.
11. Crossley, M., Lorenzetti, F.D., Naskar, S., O'Shea, M., Kemenes, G., Benjamin, P.R., and Kemenes, I. (2019). Proactive and retroactive interference with associative memory consolidation in the snail *Lymnaea* is time and circuit dependent. Commun Biol. *26*, 2:242.
12. Bargmann, C.I., Hartwieg, E., and Horvitz, H.R. (1993). Odorant-selective genes and neurons mediate olfaction in *C. elegans*. Cell *74*, 515–527.
13. Troemel, E.R., Kimmel, B.E., and Bargmann, C.I. (1997). Reprogramming *elegans*. Cell *91*, 161–169.
14. Tsunozaki, M., Chalasani, S.H., and Bargmann, C.I. (2008). A behavioral switch: cGMP and PKC signaling in olfactory neurons reverses odor preference in *C. elegans*. Neuron *59*, 959–971.
15. Carelli, R.M., and West, E.A. (2014). When a good taste turns bad: Neural mechanisms underlying the emergence of negative affect and associated natural reward devaluation by cocaine. Neuropharmacology *76*, 360–369.
16. Varela, N., Gaspar, M., Dias, S., and Vasconcelos, M.L. (2019). Avoidance response to CO_2 in the lateral horn. PLoS Biol. *17*, e2006749.
17. Dolan, M.J., Belliart-Guérin, G., Bates, A.S., Frechter, S., Lampin-Saint-Amaux, A., Aso, Y., Roberts, R.J.V., Schlegel, P., Wong, A., Hammad, A., et al. (2018). Communication from learned to innate olfactory processing centers is required for memory retrieval in *Drosophila*. Neuron *100*, 651–668.e8.
18. Schultzhaus, J.N., Saleem, S., Iftikhar, H., and Carney, G.E. (2017). The role of the *Drosophila* lateral horn in olfactory information processing and behavioral response. J. Insect Physiol. *98*, 29–37.
19. Grillner, S., Hellgren, J., Ménard, A., Saitoh, K., and Wikströmet, M.A. (2005). Mechanisms for selection of basic motor programme. Trends Neurosci. *28*, 364–370.
20. Benjamin, P.R., Staras, K., and Kemenes, G. (2010). What roles do tonic inhibition and disinhibition play in the control of motor programs? Front. Behav. Neurosci. *4*, 30.
21. Kemenes, G., and Benjamin, P.R. (1989). Appetitive learning in snails shows characteristics of conditioning in vertebrates. Brain Res. *489*, 163–166.
22. Staras, K., Kemenes, G., and Benjamin, P.R. (1999). Electrophysiological and behavioral analysis of lip touch as a component of the food stimulus in the snail *Lymnaea*. J. Neurophysiol. *81*, 1261–1273.
23. Pirger, Z., Laszlo, Z., Kemenes, I., Toth, G., Reglodi, D., and Kemenes, G. (2010). A homolog of the vertebrate pituitary adenylate cyclase-activating associative memory in an invertebrate. J. Neurosci. *30*, 13766–13773.
24. Kemenes, G., and Benjamin, P.R. (1994). Training in a novel environment improves the appetitive learning performance of the snail, *Lymnaea stagnalis*. Behav. Neural Biol. *61*, 139–149.
25. Staras, K., Kemenes, G., and Benjamin, P.R. (1998). Neurophysiological correlates of unconditioned and conditioned feeding behavior in the pond snail *Lymnaea stagnalis*. J. Neurophysiol. *79*, 3030–3040.
26. Kemenes, G., Staras, K., and Benjamin, P.R. (1997). *In vitro* appetitive classical conditioning of the feeding response in the pond snail *Lymnaea stagnalis*. J. Neurophysiol. *78*, 2351–2362.
27. Benjamin, P.R. (2012). Distributed network organization underlying feeding behavior in the mollusk *Lymnaea*. Neural Syst. Circuits *2*, 4.
28. Syed, N.I., and Winlow, W. (1991). Coordination of locomotor and cardiorespiratory networks of *Lymnaea stagnalis* by a pair of identified interneurones. J. Exp. Biol. *158*, 37–62.

STAR★METHODS

KEY RESOURCES TABLE

REAGENT or RESOURCE	SOURCE	IDENTIFIER
Chemicals, Peptides, and Recombinant Proteins		
Sucrose	Thermo Fisher Scientific	CAS: 57-50-1
Protease Type XIV	Sigma-Aldrich	P5147
Lucifer Yellow dilithium salt	Sigma-Aldrich	L0259
Experimental Models: Organisms/Strains		
Lymnaea stagnalis	University of Sussex	N/A
Deposited Data		
		https://doi.org/10.25377/sussex.13664207
Software and Algorithms		
ImageJ	NIH	https://imagej.nih.gov/ij/
Graphpad Prism version 9	Graphpad Software	https://www.graphpad.com/
pClamp version 8.2	Molecular Devices	https://www.moleculardevices.com/
Origin version 8.5	OriginLab	https://www.originlab.com/

RESOURCE AVAILABILITY

Lead contact

Further information and requests should be directed to and will be fulfilled by the Lead Contact (Ildiko Kemenes; i.kemenes@sussex.ac.uk)

Materials Availability

This study did not generate new unique reagents.

Data and Code Availability

Original data has been deposited to FigShare (https://doi.org/10.25377/sussex.13664207). This study did not generate any computer code.

Experimental Model and Subject Details

In this study we used *Lymnaea stagnalis* from a laboratory-bred stock of adult (5-6 months old) snails. Animals were kept in groups in large holding tanks containing copper-free water at 20-21°C on a 12:12 h light-dark regime. The animals were fed lettuce three times and a vegetable-based fish food (Tetra-Phyll; TETRA Werke, Melle, Germany) twice a week. Before starting an experiment, animals were food-deprived for two days.

METHOD DETAILS

Multi-trial aversive classical conditioning

Snails were trained using a multi-trial aversive classical conditioning protocol in which the order of the two stimuli (touch and sucrose) originally used for reward conditioning[19-23] was reversed and the concentration of sucrose was reduced while the intensity and number of tactile stimuli used in each trial was increased. Thus, in this paradigm, in each trial, 0.34% sucrose (final concentration in 100 mL copper-free water), the conditioning stimulus (CS), was paired with a series of seven aversive tactile stimuli, each serving as an unconditioned stimulus (US).

Before aversive training, the snails were placed individually into Petri dishes containing 95 mL of copper-free water for a 10 min acclimatization period, so that a low level of spontaneous rasping was reached in the novel environment[24]. During each trial of the tactile classical conditioning protocol, the snails were first presented with the CS. After 15 s (when all the freely moving snails had started feeding in response to the presentation of sucrose), the first of a series of seven tactile stimuli was presented using a hand-held probe with a tip made of a toothpick. Each of the individual tactile stimuli delivered with the tooth-pick probe induced a very similar withdrawal response to what we saw when we used a 4 g Von Frey hair in a previous study[5]. The target zone on the lip structure was the median portion adjacent to the mouth parts including the leading edge of the lips as previously described by Staras et al.[22]. During the rest of the 2 min trial period, the procedure was repeated every 15 s so the time of overlap between the

presence of the CS and the delivery of the seven tactile stimuli was 90 s during a 120 s-long application of the CS. The pairing of sucrose with a series of a total of 7 tactile stimuli constituted one trial. After each trial, the animals were rinsed in a clean water tank to remove any residual sucrose before they were placed back into their home tank. There was a total of five trials with a 60 min intertrial interval (ITI) (Figure 1A). For explicitly unpaired control, the snails also received five trials with a 60 min ITI. Each unpaired trial consisted of the presentation of the CS and a series of 7 aversive tactile US delivered at 15 s intervals, with 10 min intervals between the start of the application of the CS and the first tactile stimulus, in contrast to the 15 s intervals used in the paired training protocol. Similar to the paired training protocol, the snails were placed individually into Petri dishes for a 10 min acclimatization period and presented with the CS in the 0.34% final concentration, but this was not followed by the presentation of the series of US. After a 2 min period, the animals were transferred to another Petri dish (100 mL copper-free water) for 10 min, after which the US was presented 7 times with 15 s intervals. Thereafter, the animals were put back into their home tank. CS tests were performed both before and after paired and unpaired training (Figure 1A).

To test the possibility of non-associative learning influencing the post-training response to sucrose, we used three further control groups of animals. A CS alone group of animals only received 5 applications of the CS, whereas a US alone group only received 5 series of 7 tactile stimuli each, following the same temporal pattern as the CS and US applications, respectively, in the CS+US paired group. Finally, a random US alone control group received the same number (5) of series of 7 tactile stimuli as the paired and unpaired group but at random intervals.

Twenty-four h after the first trial, individual snails were taken from their home tanks using a blind procedure and placed in Petri dishes for testing the response to the CS. After a 10 min acclimatization period, rasps were counted for 2 min (i.e., spontaneous rasping in water). Sucrose was then applied, and rasps were counted for a further 2 min (i.e., the feeding response to the sucrose CS). For testing the integrity of the feeding network of trained animals as well as the stimulus specificity of the conditioned response after aversive training with sucrose and strong touch, an additional control experiment was performed. In this experiment the number of rasps was counted after presenting filtered cucumber juice to the animals as a feeding stimulus other than sucrose.

Two naive control groups of animals were also used in the experiments; both of these groups were kept in the same general conditions as the experimental ones. Animals in one of these groups (Naive 1) were only tested once with the CS at the same time when the experimental groups were tested 24 h after the paired or unpaired training. Animals in a second group (Naive 2) were tested with the CS both 1 h before and 24 h after the trials commenced in the other groups. A detailed comparison of all the training paradigms is shown in Figure S1.

Sensitization training

The pre-training treatment of the snails (n = 24) in this experiment was the same as described for the aversive conditioning experiment. However, in the pre-training test, the animals were presented with a weak tactile stimulus to the right tentacle using a hand-held probe with a tip made of a thin wedge of soft, flexible plastic[22], while their behavior was recorded by the video camera of an Apple iPhone 6S+.

During the 2-min training periods, the same strong serial tactile stimulation to the lip region that was used as the US in the aversive training protocol, was repeated every 15 s in each trial, but in the absence of the sucrose CS. After this, the animals were placed back into the home tank. Sixty minutes after the first trial, the animals received a second US only training trial followed by three more trials at 60 min intervals.

After 24 h, individual snails were taken for testing from their home tanks and placed in Petri dishes. After a 10-min acclimatization period, the same weak tactile stimulation that was used in the pre-tests was applied to the lip region of the animals while they were being videoed. The pre- and post-training video clips were randomized for blind quantitative analysis and the total area of the head/foot complex was measured 1 s before the stimulus and at 6 time points after the stimulus (0.5 s, 1 s, 1.5 s, 2 s, 2.5 s and 3 s) in each video clip using ImageJ software. The head/foot area was normalized to the before stimulus condition to compare changes induced by the weak test stimulus. A reduced area therefore represented a withdrawal response of the head/foot complex to the tactile stimulus and the size of this response was compared between the before sensitization and after sensitization time points.

Preparations for electrophysiology

Experiments were performed on semi-intact preparations containing the entire CNS and attached sensory regions (lips and tentacles)[5,22,25,26]. A modified semi-intact preparation, containing the buccal mass, the main feeding muscle, was used to measure rhythmic contractions induced or inhibited by sucrose.

Transducer recordings

Rhythmic buccal mass contractions in the preparations were activated or inhibited by sucrose (0.02 mM in normal saline) applied to the lips via a computer-controlled gravity-fed perfusion system. At the systems level, the sucrose induced responses were observed both on the buccal mass and on the feeding motoneurons (B3 or B4) confirming that they were generated by the feeding network. The buccal mass was attached to a force transducer (WPI FORT10 g, World Precision Instruments, Incorporation, Sarasota, USA). Muscle recordings were made by connecting the force transducer through a DigiData 1320A interface (Axon Instruments, Union City, CA, USA) to a PC.

Electrophysiology recordings

Preparations were dissected and neurons recorded in a Sylgard-lined chamber containing normal snail saline (50 mM NaCl, 1.6 mM KCl, 3.5 mM $CaCl_2$, 2.0 mM $MgCl_2$, 10 mM HEPES, pH 7.9). The outer layer of the thick connective tissue sheath was removed mechanically from the dorsal surface of ganglia and the inner layers were softened by 1% protease treatment (Sigma XIV, Sigma) for 2 min.

Intracellular recordings were performed under a stereomicroscope (Leica MZ FLIII, Switzerland). AxoClamp 2B (Axon Instruments, Union City, CA, USA) and NeuroLog D.C. (Digitimer Ltd., UK) amplifiers were used to monitor the electrical activity of identified neurons. Membrane potential (MP) manipulation was carried out by current injection through the recording electrode. Microelectrodes were pulled from borosilicate glass pipettes (GC200F-15, Harvard Apparatus, UK) with Narishige (Narishige Scientific Instrument Laboratory, Japan) vertical puller to a 15–20 MΩ tip resistance when filled with 4 M potassium acetate. For data acquisition and protocols, the amplifiers were connected via a DigiData 1320A interface (Axon Instruments, Union City, CA, USA) to a PC supplied with pClamp8.2 software (Axon Instruments, Union City, CA, USA). The recorded traces were analyzed by OriginLab Corporation Origin 8.5 software.

Identification of neurons

The left and right B3 feeding motoneurons of the buccal ganglia were mainly used to monitor the central pattern generator (CPG)-driven fictive feeding rhythm. In some experiments B4 feeding motoneurons of the buccal ganglia were recorded to confirm the occurrence of feeding cycles. Both types of neuron can be identified by size, location and characteristic fictive feeding activity[7,25,27].

The PIB (Pleural-Buccal) neuron is an extrinsic modulatory interneuron that inhibits the feeding network[4]. It is a small neuron (20-30 μm cell body diameter) that lies on the medial surface of the pleural ganglion close to the pleural-pedal connective[4,5]. It shows characteristic tonic firing activity and its identification is confirmed by recording inhibitory synaptic responses on feeding neurons.

PeD12 is a recently described interneuron (60 μm in cell body diameter) of the whole-body withdrawal network and its artificial stimulation excites the PIB monosynaptically causing inhibition of ongoing feeding[5]. It lies close to the previously described PeD11[28] on the dorsal surface of the pedal ganglia close to the statocyst.

Dye-injection and photoinactivation

To photoinactivate the PIB cells, they were filled with the fluorescent dye Lucifer Yellow dilithium salt (10 mM, Sigma, UK). After identification of PIB, the dye was loaded into the cell bodies from the recording microelectrode by a 10 ms and 5-8 psi pulse of a multi-channel picospritzer (General Valve Corporation, New Jersey, USA). The loaded PIB cells were photoinactivated by continuous high- energy UV flash lamp pulses using a JML-C2 (Rapp OptoElectronic, Hamburg, Germany). During photoinactivation, 1000 μs (C3 capacitor mode) 180-200 J energy pulses were used for up to 8 min. The JML-C2 was triggered by an external TTL signal. The membrane potential of PIB was monitored continuously during the flash photolysis procedure by pClamp8.2 software (Axon Instruments, Union City, CA, USA).

QUANTIFICATION AND STATISTICAL ANALYSIS

All the behavioral and electrophysiological data passed the Kolmogorov-Smirnov test for normality and therefore parametric statistical tests were used. In both the behavioral and *in vitro* experiments comparisons between more than two independent groups (e.g., naive, unpaired, trained) were carried out using ANOVA followed by multiple post hoc Tukey's tests. When comparing only two independent samples, unpaired t tests were used. When comparing two sets of data obtained under different conditions from the same group of animals, paired t tests were used. When comparing time-dependent changes in the same group of animals under two different conditions, a two-way ANOVA with RM (repeated-measures) was used, followed by paired t tests. All statistical analyses were carried out using Prism (GraphPad) software. The differences were considered statistically significant at $p < 0.05$. Group numbers and details of the statistical tests can be found in the figure legends.

Current Biology, Volume *31*

Supplemental Information

Interneuronal mechanisms for learning-induced switch in a sensory response that anticipates changes in behavioral outcomes

Zsolt Pirger, Zita László, Souvik Naskar, Michael Crossley, Michael O'Shea, Paul R. Benjamin, György Kemenes, and Ildikó Kemenes

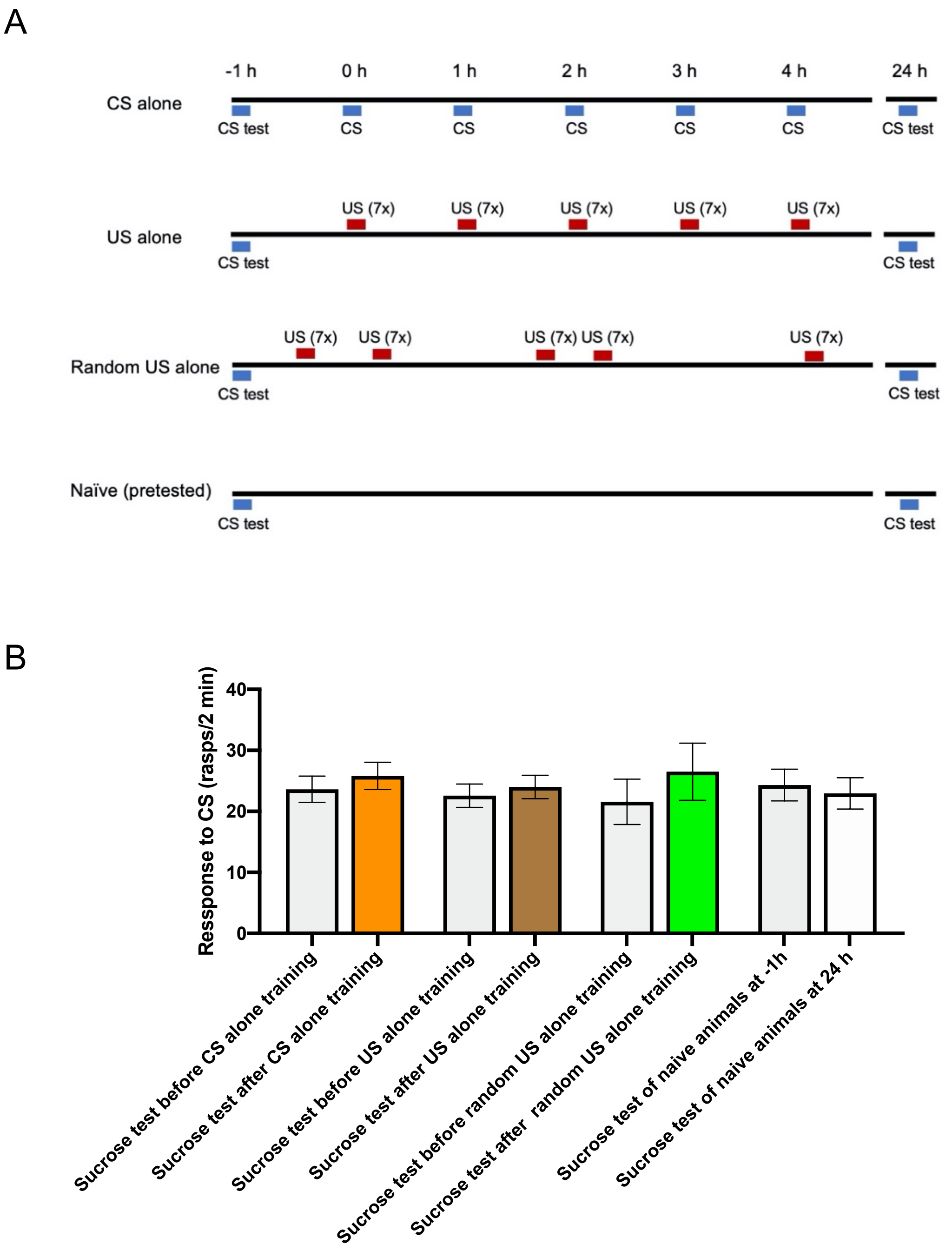

Figure S1. Non-associative training protocols do not reduce the innate behavioural response to a food stimulus. Related to Figure 1. A. The experimental groups and protocols used in the experiments. In each of the 5 CS (conditioned stimulus) alone trials only sucrose was given to the animals, in each of the 5 US (unconditioned stimulus) alone and random US alone trials, respectively, only a series of 7 strong tactile stimuli were delivered at 15 s intervals to the head of the animals. B. After the non-associative protocols, neither group shows a significantly reduced feeding response to sucrose compared to their responses before training (unpaired t-tests, each p value > 0.05). The naïve group that was pre-tested with sucrose at the same time as the other three groups (-1h) does not show a change in its response at 24h either. Graphs show means ± standard error of means (SEM). ANOVA for the CS alone, US alone, random US alone and Naïve 24 h test data: $F[3, 56] = 0.31$, $p = 0.82$ (n.s.).

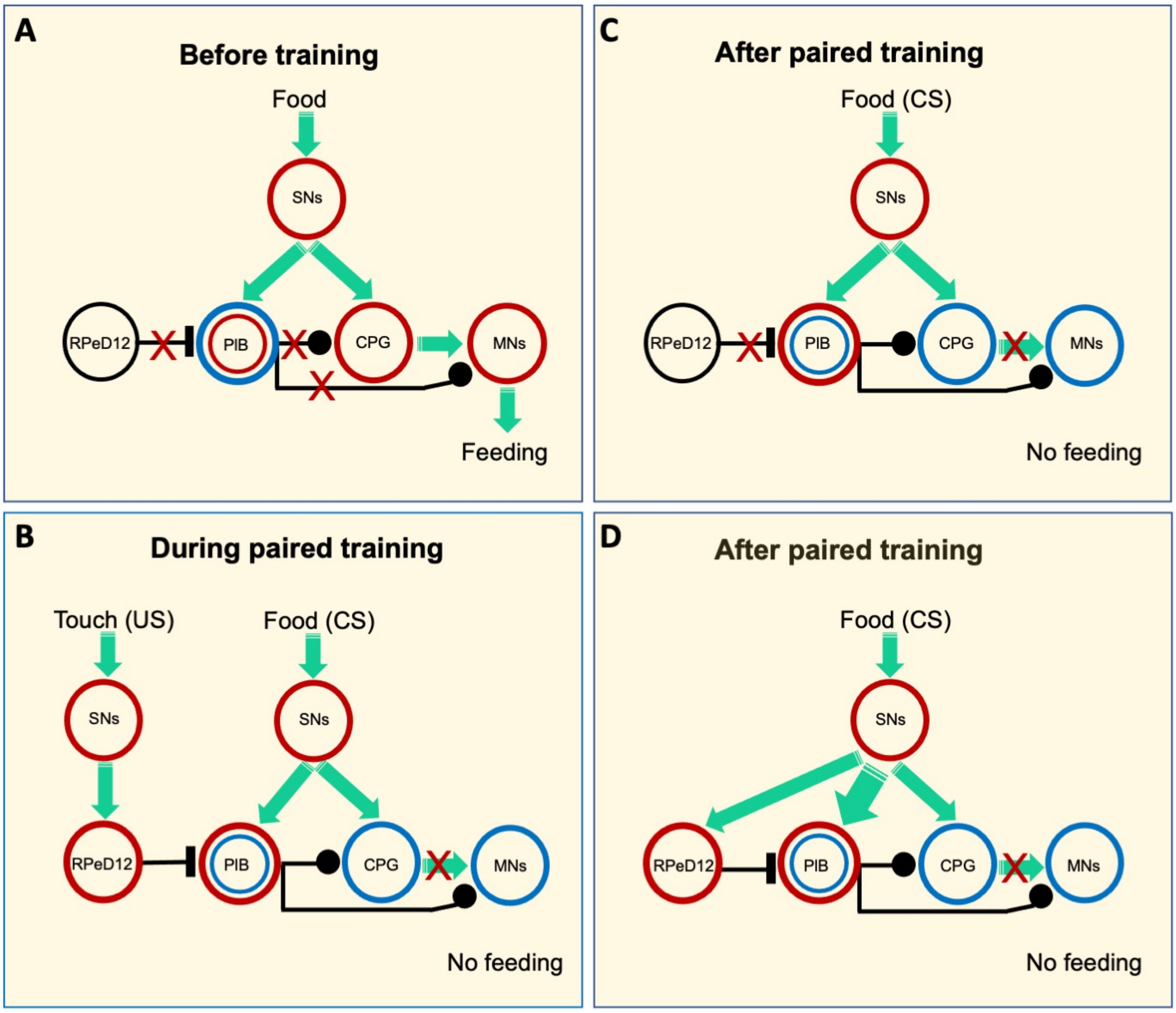

Figure S2. Simplified circuit diagrams of possible interneuronal mechanisms of conditioned inhibition of feeding by sucrose. Related to Figure 3.

A. As indicated by the red outlines, in naïve animals (before training), sucrose, a powerful food stimulus, excites the feeding central pattern generator (CPG) network[S1], which in turn activates motoneurons (MNs) of the feeding system that generate the three-phase feeding motor pattern[S2]. In the absence of the sucrose stimulus, the pleural-buccal interneuron PIB has a tonic inhibitory control over the feeding network, including the CPG and motoneurons[S3]. This tonic inhibition is temporarily suppressed (red crosses) due to the hyperpolarizing effect of the food stimulus on this interneuron (see Figures 2A and 2B), indicated by the blue outline of the circle representing PIB. The smaller red circle inside the blue one indicates the baseline level of PIB membrane potential/firing rate in naïve animals before the application of sucrose. The identified right pedal 12 withdrawal interneuron (RPeD12), which forms a strong chemical excitatory monosynaptic connection with PIB[S4], does not play a role in the circuit events of food-induced activation of the feeding motor program (indicated by the black outline of the circle representing RPeD12 and the crossed out black line with the solid black rectangle).

B. During paired training with sucrose as the CS and multiple series of strong tactile stimuli as US (see Figure 1A), RPeD12 is strongly excited by the touch US, as indicated by the red outline of the circle representing this interneuron. In turn, RPeD12 excites PIB, which will then inhibit the feeding network. The smaller blue circle indicates the hyperpolarized level of PIB membrane potential in naïve animals during the application of sucrose (the food CS) in each

trial (see Figure 1A), whereas the red outline indicates the excitation of PIB by the US-activated RPeD12. The strong excitatory input from RPeD12 to PIB overcomes the background inhibition by the sucrose-driven sensory input and results in the net depolarization of PIB. Note that potential sites of CS - US co-incidence detection in this circuit are the PIB and the CPG. However, it cannot be ruled out that terminals of the CS sensory pathway presynaptic to PIB can also be modulated during paired training.
C. One hypothetical scenario is that after paired training, there is a learning-induced plastic change in the electrical properties of PIB, for example, a persistent hyperpolarization of its baseline membrane potential level before the application of sucrose, indicated by the smaller blue circle within the red circle representing the PIB depolarized by the CS. The shift of the PIB mebrane potential to a more negative level compared to the naïve level may lead to the pre-training hyperpolarizing response to sucrose (indicated by the blue outline in A) being reversed into a depolarizing one (indicated by the red outline in C), removing the suppression of PIB's inhibitory effect on the feeding network.
D. One alternative hypothesis is that after paired training, the now aversive conditioned food stimulus sucrose also excites RPeD12, which in turn further excites PIB, which prevents the activation of the feeding network. This latter hypothesis is based on the finding that in naïve animals RPeD12 is strongly excited by aversive tactile stimulation of the head, so it seemed possible that when, after paired training, the initially strongly appetitive sucrose stimulus evokes a strong aversive response in the feeding system, this is mediated by RPeD12. We cannot rule out either that plastic changes leading to a persistent strengthening of the CS to PIB pathway (thicker green arrow connecting the sensory neurons (SNs) to PIB) also contribute to the sucrose-induced excitation of PIB after training.

The simplified diagrams in this figure only show the identified monosynaptic excitatory (black lines with solid squares) and inhibitory (black lines with solid circles) connections between RPeD12 and PIB[S4], and PIB and the CPG as well as the feeding motoneurons[S3], respectively. All other known and putative direct or indirect inputs are indicated by the green arrows.

Supplemental References

S1. Kemenes, G., Staras, K., and Benjamin, P.R. (2001) Multiple types of control by identified interneurons in a sensory-activated rhythmic motor pattern. J Neurosci *21*, 2903-2911.

S2. Rose, R.M., and Benjamin, P.R. (1979). The relationship of the central motor pattern to the feeding cycle of *Lymnaea stagnalis*. J Exp Biol *80*, 137-163.

S3. Alania, M., Sakharov, D.A., and Elliott, C.J. (2004). Multilevel inhibition of feeding by a peptidergic pleural interneuron in the mollusc *Lymnaea stagnalis*. J Comp Physiol A Neuroethol Sens Neural Behav Physiol *190*, 379-390.

S4. Pirger, Z., Crossley, M., Laszlo, Z., Naskar, S., Kemenes, G., O'Shea, M., Benjamin, P.R., and Kemenes, I. (2014). Interneuronal mechanism for Tinbergen's hierarchical model of behavioral choice. Curr Biol *24*, 2018-2024.
